# Bryophytes promote the development of soil function on karst rock surfaces

**DOI:** 10.3389/fpls.2026.1725613

**Published:** 2026-03-06

**Authors:** Wen-ping Meng, You-jin Yan, Jing-cheng Ran, Hong Zhou, Xin-wei Zhou, Ting Zheng

**Affiliations:** 1 Guizhou Botanical Garden, Guiyang, China; 2 College of Forestry and Grassland, College of Soil and Water Conservation, Nanjing Forestry University, Nanjing, China; 3 Guizhou Academy of Forestry Sciences, Guiyang, China; 4 College of Agriculture, Forestry Engineering and Planning, Tongren University, Tongren, China

**Keywords:** bryophyte, ecological function, karst ecosystem, microorganisms, nutrition, organic acids

## Abstract

**Introduction:**

Bryophytes is a pioneer plant in the development of karst ecosystems and plays an important role in altering the surface environment of rocks. Studying the interaction mechanism between bryophyte and rock surface environment in karst can reveal the role of bryophyte in karst ecosystems and provide technical methods for the restoration of rocky desertification.

**Methods:**

By comparing the soil fertility, organic acids, microbial diversity, and community composition in the rock surface covered with bryophyte or without.

**Results:**

The experimental results show that when the rock surface covered with bryophyte, the overall contents of soil organic carbon (SOC) and total nitrogen (TN) was increased, whereas the contents of total phosphorus (TP) and total potassium (TK) decreased. And *Hyophila involute* can increase the content of malic acid and acetic acid in rock surface. In addition, the growth rates of various microorganisms were as follows: fungi, 52%; bacteria, 11%; eukaryotes, 78%; Archaea, 27%; and viruses, 146% in the rock surface covered with bryophyte. The number of carbon-fixing microorganisms increased by 37%, the number of nitrogen-fixing microorganisms increased by 49%, and the number of phosphorus-metabolizing microorganisms increased by 53% in the rock surface covered with bryophyte. *Acidimimicrobia_bacterium*, *Acidimimicrobiaceae_bacterium*, *Acidimimicrobiales_bacterium*, and *Iamiaceae_bacterium_SCSIO_58843* were significantly positively correlated with the potassium content in the soil. *Alphaprotoobjective_bacterium*, *Solirubrobacteriales_bacterium*, and *Betaproteobjective_bacterium* were significantly positively correlated with the succinic acid content in the soil. *Chloroflexi_bacterium* was significantly positively correlated with the oxalic acid content in the soil.

**Discussion:**

Bryophytes can increase the number of microorganisms related to nitrogen fixation, carbon sequestration, phosphorus metabolism, as well as soil nitrogen, organic carbon, and malic acid content, promoting the positive succession of rock surface ecosystems.This study makes it possible to use lithophytic bryophyte to control the bare rock of rocky desertification.

## Introduction

Bryophytes provide critical ecosystem services, but study on them has largely been ignored until recently (Cornelissen et al., 2007). With approximately 21,000 species, bryophytes are widely distributed across all the global continents (Zhu et al., 2022). Bryophytes, as a non-vascular plant, have developed a unique variety of physiological and morphological strategies (Wood, 2007; Zhao et al., 2015), such as desiccation tolerance, i.e., the ability to dry to equilibrium with moderate to extremely dry air and to recover the normal metabolic functions after rehydration that allow them to survive in extreme habitats such as the Sahara, Mojave, or Atacama deserts (Stark and Whittemore, 2000; Warren-Rhodes et al., 2007). Therefore, bryophytes have strong ecological adaptability and can survive in various ecosystems (Wang et al., 2026). A kind of bryophytes growing on the rock surface are called lithophytic bryophytes (Meng et al., 2021a). There are rich species of lithophytic bryophytes in karst areas, which have strong tolerance to drought and have a high calcium level (Meng et al., 2023). Lithophytic bryophytes can regulate the microclimate of a rock surface and can also reduce the soil erosion on the rock surface (Shen et al., 2018; Tu et al., 2021). Bryophytes can delay the evaporation time of rock surface water and increase the time of rock hydrochemistry. For bryophytes on the rock surface, the evaporation water loss and active water absorption time were 48% and 57% longer than those of bare rock, respectively, and the water holding capacity was increased by 16.6 times (Cao and Yuan, 1999). The water holding capacity of *Hyophila involuta* was the highest (581.71%); those of *Barbula unguiculata* and *Bryum paradoxum* were 408.88% and 389.82%, respectively (Xie et al., 2025). Moreover, *Barbula unguiculata* and *Bryum paradoxum* showed stronger resistance to high temperature, acid and alkali, and erosion (Xie et al., 2025). Under the 30° slope, the soil erosion amount (71.1 g, 67.2 g) of *Barbula unguiculata* and *Bryum paradoxum* decreased by more than 62% compared with bare soil (187.8 g) (Xie et al., 2025). The metabolic secretions and H_2_CO_3_ produced by respiration of lithophytic bryophytes can react with minerals in the rock. This process can destroy the mineral crystal structure, lead to mineral cracking, make the rock surface collapse and fall off, change the rock surface morphology, shape the karst landform, and form the original soil (Meng et al., 2019). These studies indicate that lithophytic bryophytes promote the positive development of karst ecosystems.

One of the most difficult ecological problems in karst areas is rocky desertification (Wang, 2003). Karst forest ecosystems have been affected by natural factors and human disturbance, vegetation has been destroyed, soil erosions have occurred, land productivity has been reduced, and there have been large areas of exposed rock, thus forming rocky desertification (Luo et al., 2021). Vascular plants are difficult to survive on bare rock surfaces. Therefore, the ecological restoration of bare rock surfaces is the most difficult point about rocky desertification control. However, lithophytic bryophytes can grow on the rock surface. Although they are small, their biomass in rocky desertification areas is not low. Some studies have found that there are 63 species of lithophytic bryophytes belonging to 13 families in rocky desertification areas (Meng et al., 2021b). The average biomass of bryophytes in some rock surface microhabitats, such as small rock pits, rock fissures, and rock gullies, were 0.0033, 0.0011, and 0.00097 g/cm^2^, respectively (Meng et al., 2021b). The biomass of *Bryum algovicum* was the highest at 4,508 kg/hm^2^ (Dai et al., 2021). Bryophytes can increase the roughness of the soil surface by 29.11% and reduce surface runoff and soil erosion by 70.7% and 99.2%, respectively (Zeng et al., 2025). Therefore, the study of lithophytic bryophyte improvement and restoration of the environment of rock surfaces will be a new breakthrough about rocky desertification control. This study compares the differences in carbon content, nitrogen content, phosphorus content, organic matter content, organic acid content, microbial richness, community composition, and microbial function between lithophytic bryophytes and bare rock surfaces. This study shows whether lithophytic bryophytes have the ability to improve the ecological environment about the rock surface. This study provides scientific and theoretical support for ecological control of rocky desertification bare rock.

## Materials and methods

### Sample collection and processing

**Figure 1:**
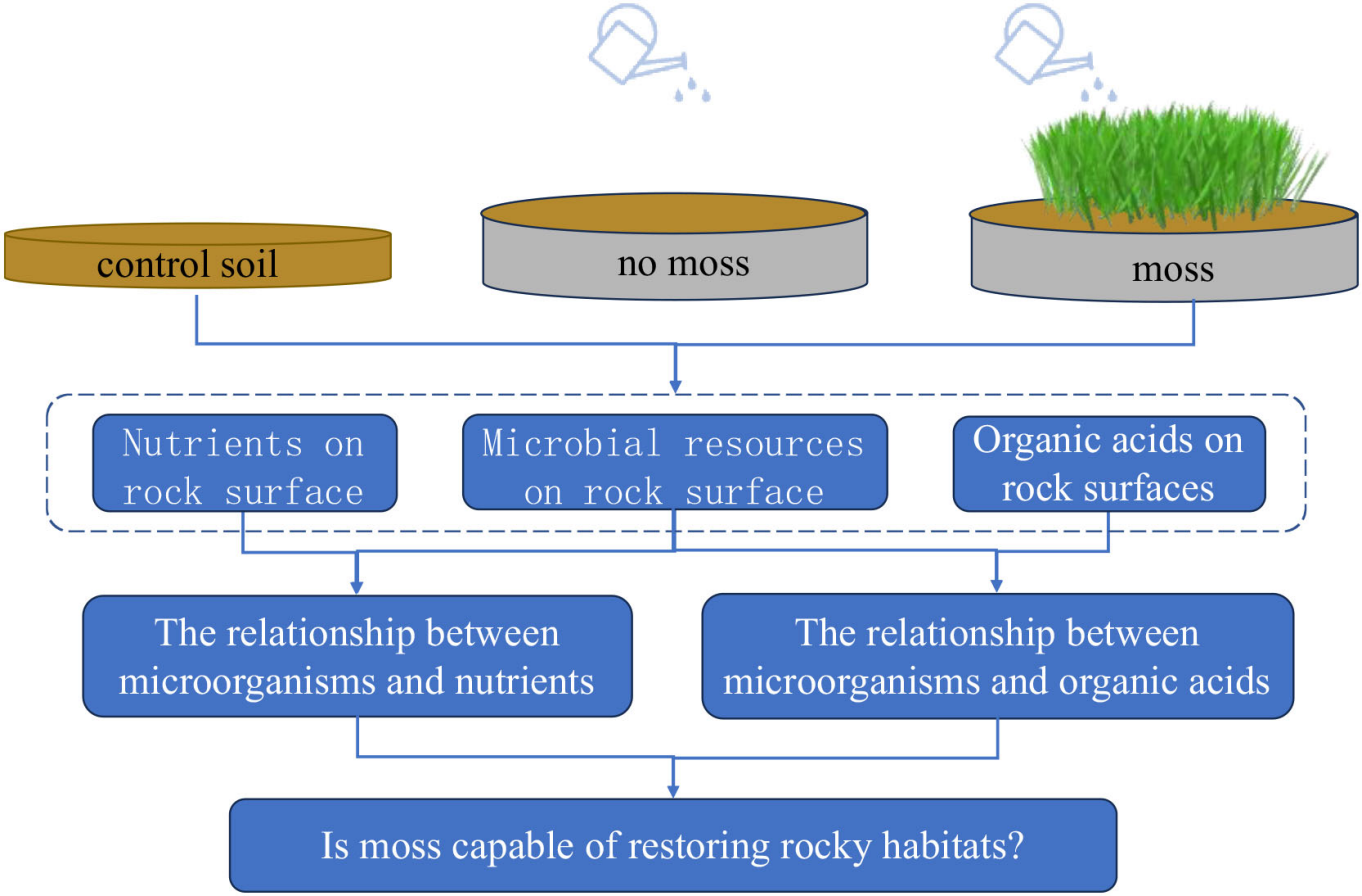
Experimental process diagram.

Three lithophytic bryophytes were covered on the rock surface as the experimental group and compared with bare soil habitat and original soil (**Figure 1**). A 1-mm-thick layer of soil was laid on the surface of a carbonate rock to simulate the habitat of lithophytic bryophytes in natural environments. Then, *Eurohypnum leptothallum*, *Didymodon constrictus*, and *Hyophila involuta* were planted on the carbonate rock surface. The rock surface without bryophyte was the bare soil as control. The experimental group and the control were set up with three replicate treatments. From the soil on the rock surface, three samples were taken as the original control soil. All the experimental groups were placed in a climate incubator with a daytime temperature of 25°C, a 12-h cycle, a light intensity of 5,000 lx, a relative humidity of 85%, and a nighttime temperature of 18°C, with a 12-h cycle, a light intensity of 0 lx, and a relative humidity of 85%. Demineralized water was sprayed on the experimental group once a week with a fixed amount of water. The pH of the water was 7. The experimental period was 12 months. After 12 months, the soil on the surface of the stone tablets was collected. After 12 months, the soil at the underside of the bryophytes and the leaching experimental group were collected. Impurities were removed, and the soil samples were quickly frozen in liquid nitrogen and stored in a −80 °C refrigerator to test the total nitrogen, total phosphorus, total potassium, organic carbon, and organic acid contents; microbial diversity; and metagenomic composition.

### Soil fertility testing

We used the potassium dichromate external heating method to determine the soil organic carbon (SOC) content, the Kjeldahl method to determine the total nitrogen content (TN), the perchloric acid–sulfuric acid digestion and molybdenum antimony colorimetric method to determine the total phosphorus (TP) content, and the hydrofluoric acid–perchloric acid digestion method to determine the total potassium (TK) content.

### Soil organic acid content testing

High-performance liquid chromatography was used to detect oxalic acid, acetic acid, malic acid, malonic acid, citric acid, and succinic acid in soil. (1) Mixed-label configuration was performed. The aforementioned six organic acids were weighed at appropriate amounts accurately and diluted with ultrapure water. Then, the solution was filtered through a microporous membrane with a pore size of 0.45μm, transferred to a 50-mL volumetric flask, diluted to a volume, and stored in a refrigerator at 4 °C. (2) A standard curve was drawn. Based on the UV absorption sensitivity, a mixed standard solution was prepared by dissolving oxalic acid, acetic acid, malic acid, malonic acid, citric acid, and succinic acid in a ratio of 0.2:5:5:5:5:5. Moreover, the mixed standard solution was diluted step by step to prepare standard solutions of five concentration levels A, B, C, D, and E, in order to draw a standard curve. The concentration of the A-level standard solution was 0.2:5:5:5:5:5 (μg/mL); dilution concentrations of B, C, and D were five times the previous level concentration, and that of E was twice that of D. (3) The chromatographic conditions were as follows: reversed-phase C18 column CAPCELL PAK C18 MG 4.6 mm × 250 mm, 5 μm, pH range: 2~10; mobile phase: deionized water containing 0.1% phosphoric acid and acetonitrile (V/V) in a ratio of 98:2; detector wavelength: 210 nm, flow rate: 1 mL/min, injection volume: 20 μL, column temperature: 35 °C. (4) Soil sample processing was performed. In each experimental group, three 5-g soil samples were weighed and placed into a 25-mL centrifuge tube. To this, 10 mL of 0.1% H_3_PO_4_ aqueous solution was added. The mixture was shaken for 1 min and centrifuged at 5,000 r/min for 5 min; the supernatant was filtered through a 0.45-μm filter membrane; and instrument testing was performed.

### Soil microbial diversity and metagenomic detection

#### DNA extraction

Total genomic DNA was extracted from the soil samples using the Mag-Bind^®^ Soil DNA Kit (Omega Bio-tek, Norcross, GA, U.S.) according to the manufacturer’s instructions. The concentration and purity of the extracted DNA were determined with TBS-380 and NanoDrop 2000, respectively. The quality of the extracted DNA was assessed on 1% agarose gel.

### Library construction and metagenomic sequencing

The extracted DNA was fragmented to an average size of approximately 400 bp using the Covaris M220 system (Gene Company Limited, China) for paired-end library construction. A paired-end library was constructed using NEXTFLEX Rapid DNA-Seq (Bioo Scientific, Austin, TX, USA). Adapters containing the full complement of sequencing primer hybridization sites were ligated to the blunt ends of the fragments. Paired-end sequencing was performed on an Illumina NovaSeq instrument (Illumina Inc., San Diego, CA, USA) at Majorbio Bio-Pharm Technology Co., Ltd. (Shanghai, China), with a NovaSeq 6000 S4 Reagent Kit v1.5 (300 cycles) according to the manufacturer’s instructions (www.illumina.com).

### Sequence quality control and genome assembly

The data were analyzed on the free online platform of the Majorbio Cloud Platform (www.majorbio.com). Briefly, the paired-end Illumina reads were trimmed of adaptors, and low-quality reads (length <50 bp or with a quality value <20 or having N bases) were removed using fastp. Contigs with a length ≥300 bp were selected as the final assembly result, and the contigs were subsequently used for further gene prediction and annotation.

### Gene prediction, taxonomy, and functional annotation

Open reading frames (ORFs) from each assembled contig were predicted using Prodigal/MetaGene. The predicted ORFs with a length ≥100 bp were retrieved and translated into amino acid sequences using the NCBI translation table.

A non-redundant gene catalogue was constructed using CD-HIT with 90% sequence identity and 90% coverage. High-quality reads were aligned to the non-redundant gene catalogues to calculate gene abundance with 95% identity using SOAPaligner.

### Species and functional annotations

Representative sequences of the non-redundant gene catalogue were aligned to the NR database with an e-value cut-off of 1e^−5^ using Diamond for taxonomic annotations. Cluster of Orthologous Groups (COG) protein annotation for the representative sequences was performed using Diamond against the EggNOG database, with an e-value cut-off of 1e^−5^. The KEGG annotation was conducted using Diamond against the Kyoto Encyclopedia of Genes and Genomes database with an e-value cut-off of 1e^−5^.

### Analysis

The soil physical and chemical indicators were analyzed via Origin, and a graph was created. SPSS and Excel were used to statistically analyze the microbial diversity data and create charts. The Meiji biological cloud database was used to analyze the data, and the dominant species and functional composition of the community were studied using the column graph visualization method. The community heatmap analysis tool was used to calculate the species/gene/functional abundance of each sample. Hierarchical clustering of the distance matrix clearly reveals the distance of the sample branches. On the bases of the obtained species and functional abundance data of different groups, one-way analysis of variance was used to conduct hypothesis tests on the species and functions among their microbial communities; evaluate the significance level of species, functions, or gene abundance differences; and obtain the species and functions with significant differences between groups. The correlations between different species and soil physical and chemical indices were analyzed.

## Results

### Effects of bryophytes on the soil fertility of rock surfaces

**Figure 2:**
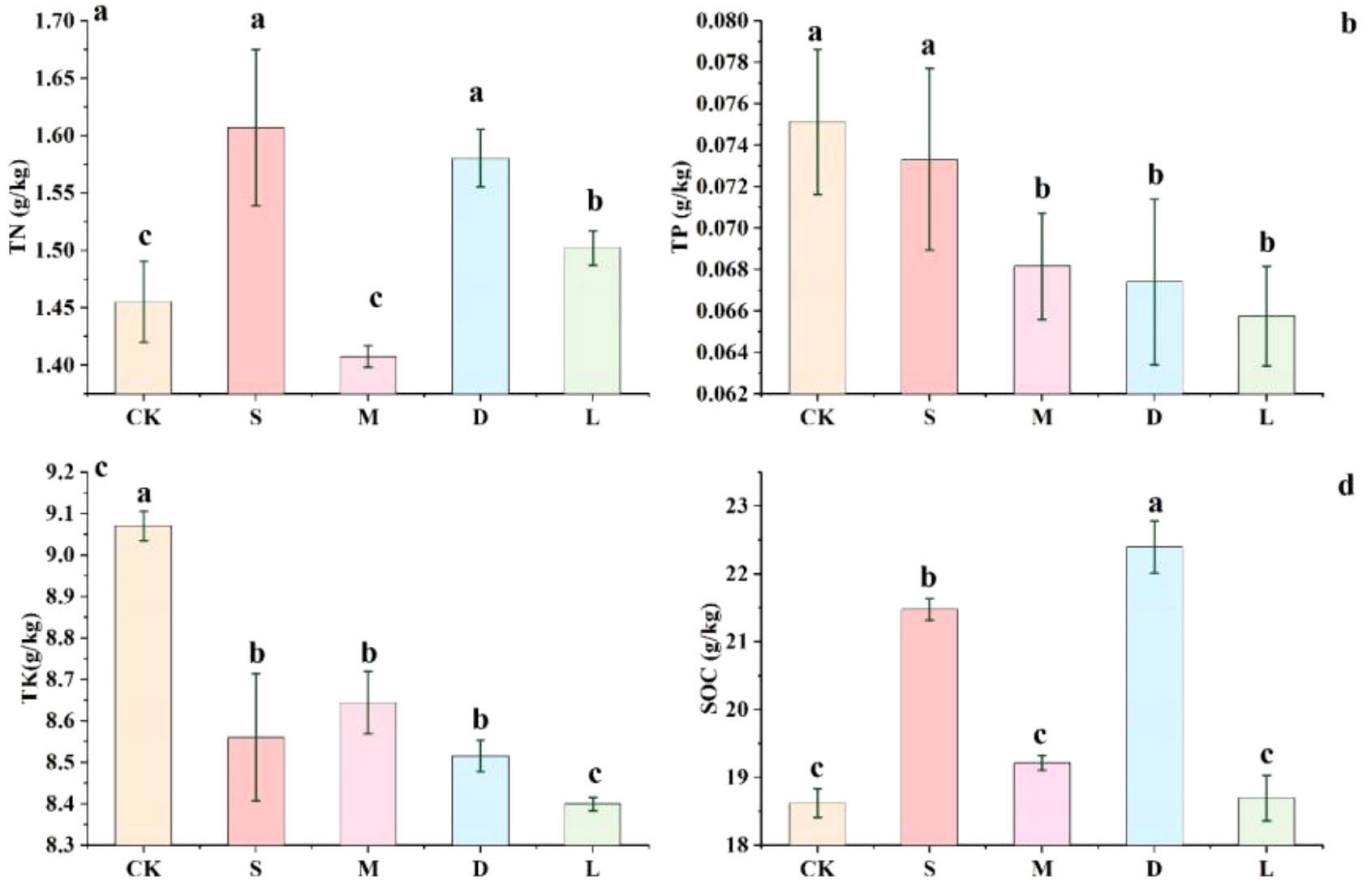
Effect of lithophytic bryophytes on soil fertility on rock surfaces. **(a)** impact of mosses on the total nitrogen content of rock surfaces; **(b)** impact of mosses on the total phosphorus content of rock surfaces; **(c)** impact of mosses on the total potassium content of rock surfaces; **(d)** impact of mosses on the organic carbon content of rock surfaces; CK: original control soil, S: *Hyophila involuta*, M: *Eurohypnum leptothallum*, D: *Didymodon constrictus*, L: bare soil.

A comparison of the changes in soil fertility between rock surfaces with lithophytic bryophytes and bare soil and original soil revealed that lithophytic bryophytes can increase the contents of soil organic carbon (SOC) and total nitrogen (TN) in rock surface habitats. However, the contents of total phosphorus (TP) and total potassium (TK) were decreased in the rock surface with lithophytic bryophytes (**Figure 2**). Moreover, there are interspecies differences. Among the three lithophytic bryophytes, *Hyophila involuta* and *Didymodon constrictus* showed the most prominent increase in TN content, and *Didymodon constrictus* showed the most prominent increase in SOC content. In addition, the decline in TP and TK content was most severe in bare soil without lithophytic bryophytes covered.

### The influence of bryophytes on organic acids on rock surfaces

**Figure 3:**
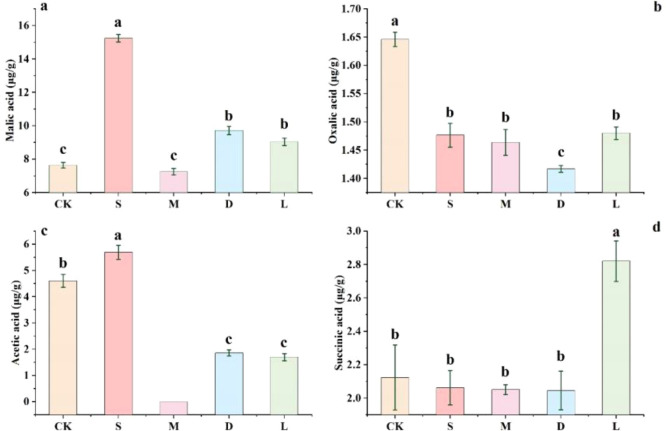
Effect of bryophytes on organic acids on rock surfaces. **(a)** impact of bryophytes on the malic acid content of rock surfaces; **(b)** impact of bryophytes on the oxalic acid content of rock surfaces; **(c)** impact of bryophytes on the acetic acid content of rock surfaces; **(d)** impact of bryophytes on the succinic acid content of rock surfaces; CK: original control soil, S: *Hyophila involuta*, M: *Eurohypnum leptothallum*, D: *Didymodon constrictus*, L: bare soil.

There are interspecific differences in the promotion of organic acid content in rocky habitats by lithophytic bryophytes (**Figure 3**). *Hyophila involuta* significantly increased the content of malic acid and acetic acid in rock surfaces. The content of succinic acid in the bare soil environment was higher than that in rocky surfaces covered with lithophytic bryophytes.

### The impact of bryophytes on the microbial diversity of rock surfaces

**Figure 4:**
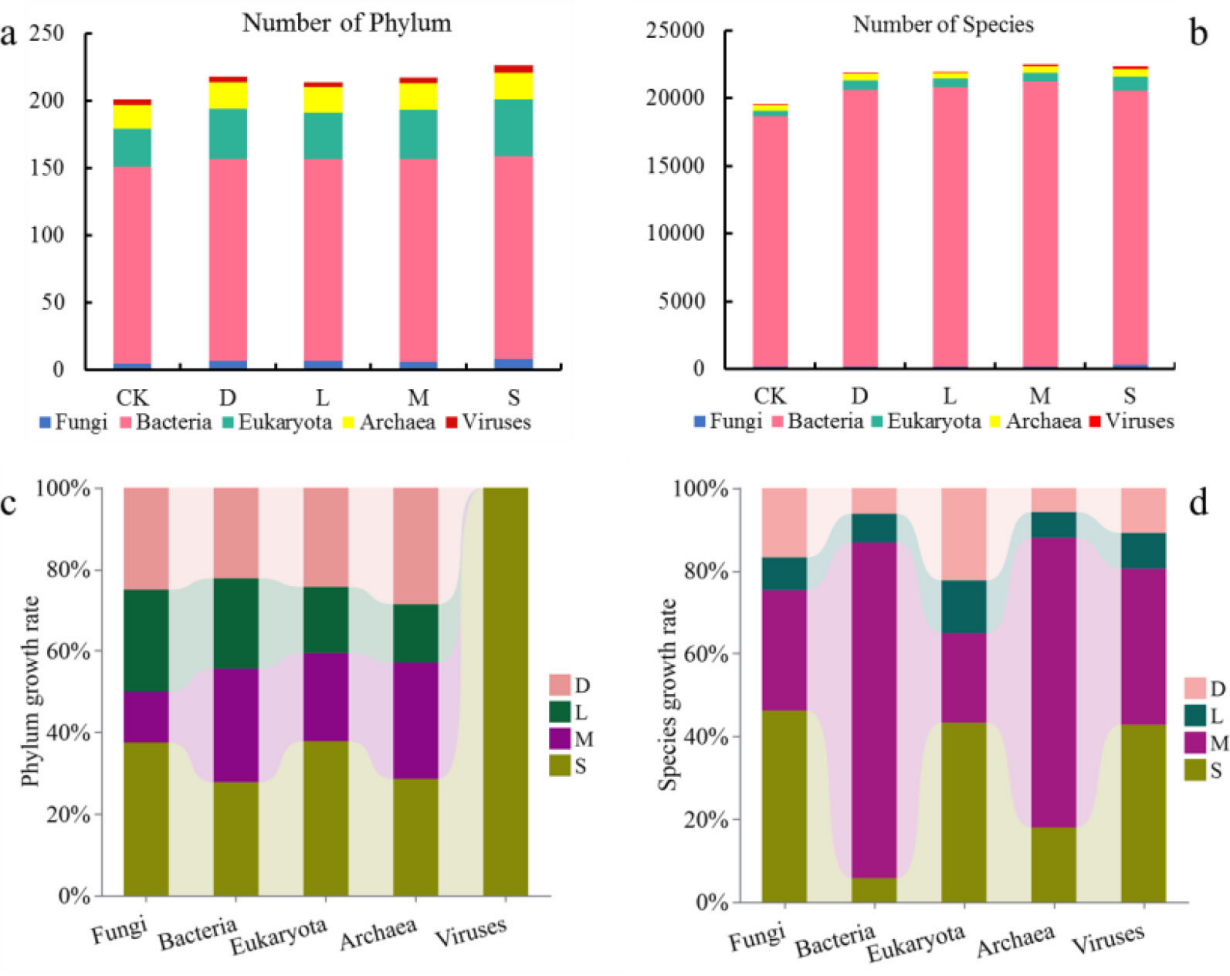
Contribution of bryophytes to microbial resources on rock surfaces. **(a)** number of microbial phylum on rock surfaces influenced by different lithophytic bryophytes; **(b)** number of microbial species on rock surfaces influenced by different lithophytic bryophytes; **(c)** proportions of fungi, bacteria, eukaryotes, archaea, and viruses on the rock surface of lithophytic bryophytes; **(d)** proportion of species about fungi, bacteria, eukaryotes, archaea, and viruses on the rock surface of lithophytic bryophytes; CK: original control soil, S: *Hyophila involuta*, M: *Eurohypnum leptothallum*, D: *Didymodon constrictus*, L: bare soil.

The average number of microorganisms on the rock surface with bryophytes was 22,340 (*Hyophila involuta* 22,063, *Eurohypnum leptothallum* 22,779, *Didymodon constrictus* 22,180), for the bare soil environment it was 22,329, and for the original control soil it was 19,925 (**Figure 4b**). Compared with the original control soil, the rock surface covered with bryophytes increased the number of microorganisms in the soil. However, the difference is not significant compared with bare soil. Also, there was no significant difference between the rock surface covered with bryophytes and bare soil.

These microorganisms include mainly fungi, bacteria, eukaryotes, archaea, and viruses. At the phylum level, bacteria had the greatest number of species, followed by eukaryotes, archaea, fungi, and viruses (**Figure 4a**). Compared with the original control soil, bare soil, and other bryophytes, *Hyophila involuta* presented the greatest increase in the number of phyla of fungi, eukaryotes, and viruses within the rocky habitat, at 60%, 50%, and 25%, respectively. The proportion of the increase in the number of phyla in Archaea caused by the three bryophytes was the same and was greater than that caused by the bare soil environment (**Figure 4c**). At the species level, compared with the original control soil, bare soil, and other bryophytes, *Hyophila involuta* had the highest proportion of increase in the number of microbial species in fungi, eukaryotes, and viruses within the rock surface, at 98.06%, 127.02%, and 244.44%, respectively (**Figure 4d**). The maximum increase in the number of microbial Bacteria and Archaea species were achieved by *Eurohypnum leptothallum*, with rates of 135% and 171.43%, respectively.

### The impact of bryophyte plants on the diversity and community composition of functional microorganisms on rock surfaces

**Figure 5:**
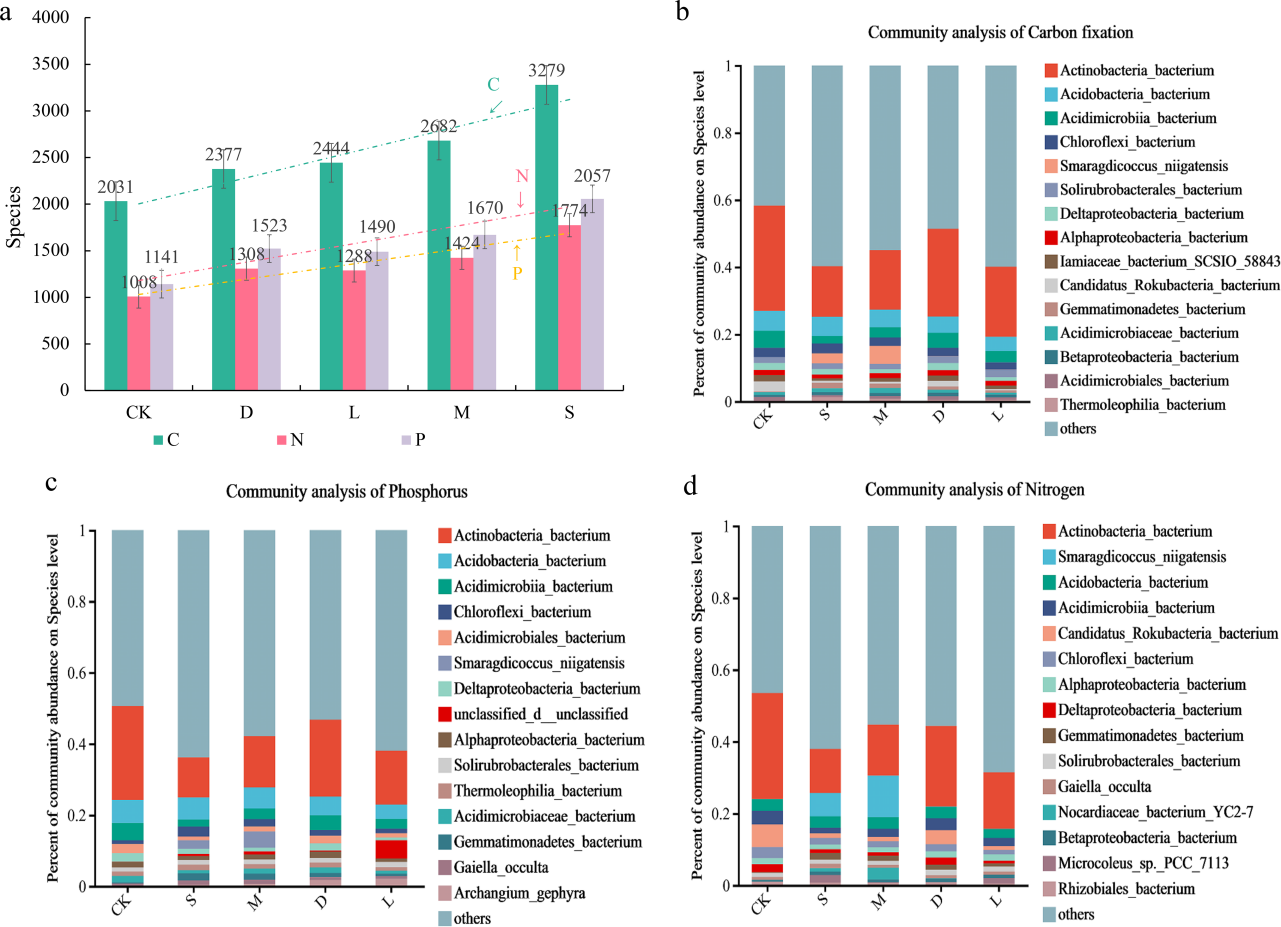
Changes of microorganisms related to carbon fixation, nitrogen fixation and phosphorus metabolism in the habitat of lithophytic bryophytes after planting on rock surfaces. **(a)** differences in the impact of different moss species on the number of carbon sequestration, nitrogen fixation, and phosphorus metabolism-related microorganisms in rock surface habitats; CK: original control soil, S: *Hyophila involuta*, M: *Eurohypnum leptothallum*, D: *Didymodon constrictus*, L: bare soil, C: number of microorganisms related to carbon fixation, N: number of microorganisms related to nitrogen fixation, P: number of microorganisms related to phosphorus metabolism. **(b)** Differences of microbial species about carbon fixation on the rock surface habitat among different moss species. **(c)** Differences of microbial species about nitrogen fixation on the rock surface habitat among different moss species. **(d)** differences of microbial species about phosphorus metabolism on the rock surface habitat among different moss species.

Compared with those in the original control soil group, the number of microbial species related to carbon fixation, nitrogen fixation, and phosphorus metabolism on the rock surface covered with bryophytes was increased. There were also differences in the impacts of different bryophyte species on the number of functional microbial species (**Figure 5**). *Hyophila involuta* had the greatest impact on the functional microbial resources, increasing the number of carbon-fixing microorganisms by 61.44%, the number of nitrogen-fixing microorganisms by 75.99%, and the number of phosphorus-metabolizing microorganisms by 80.28% in the rock surface. *Eurohypnum leptothallum* increased the number of carbon-fixing, nitrogen-fixing, and phosphorus-metabolizing microorganisms by 32.05%, 41.27%, and 46.36%, respectively. An analysis of the microbial community composition revealed that *Actinobacteria_bacterium*, *Acidobacteria_bacterium*, and *Acidimicrobiia_bacterium* were the most abundant of microorganisms in the three functional groups. Moreover, similarity was noted among the top 20 microbial species in terms of abundance ranking among the three functional groups. However, the abundance of some species varies among different functional groups. A microbial species may have multiple ecological functions. In addition, many undefined species exist in various functional groups, and they may play a significant role in improving the functionality of rock surfaces.

### Functional analysis of microorganisms on mossy rock surfaces

#### The metabolic pathways of carbon-fixing microorganisms

**Figure 6:**
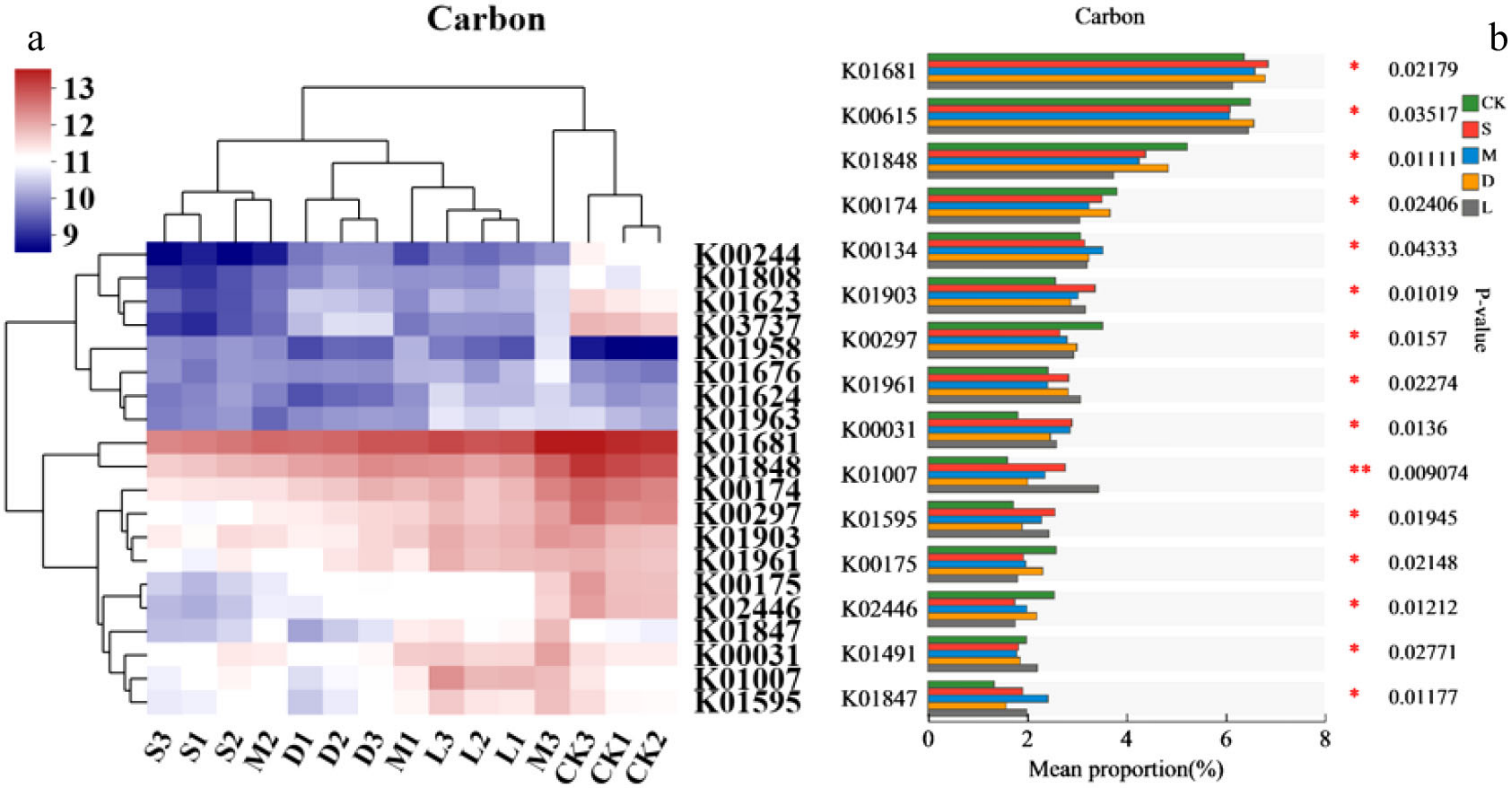
Changes in carbon fixation based on KEGG analysis of the habitat after lithophytic bryophytes were grown on the rock surface. **(a)** KEGG with the top 15 abundances; **(b)** KEGG with significant differences; CK: original control soil, S: *Hyophila involuta*, M: *Eurohypnum leptothallum*, D: *Didymodon constrictus*, L: bare soil.

There were significant differences in the metabolic pathways of the carbon-fixing microorganisms on the rock surface covered with bryophytes compared with those in the original control soil (**Figure 6a**). The abundance of metabolic pathways K01681, K01903, K01961, K00031, K01007, K01595, and K01847 in the rock surface covered with bryophytes was significantly greater than that in the original control soil. However, the abundances of K01848, K00174, K00297, K00175, and K02446 decreased significantly (**Figure 6b**).

#### The metabolic pathways of nitrogen-fixing microorganisms

**Figure 7:**
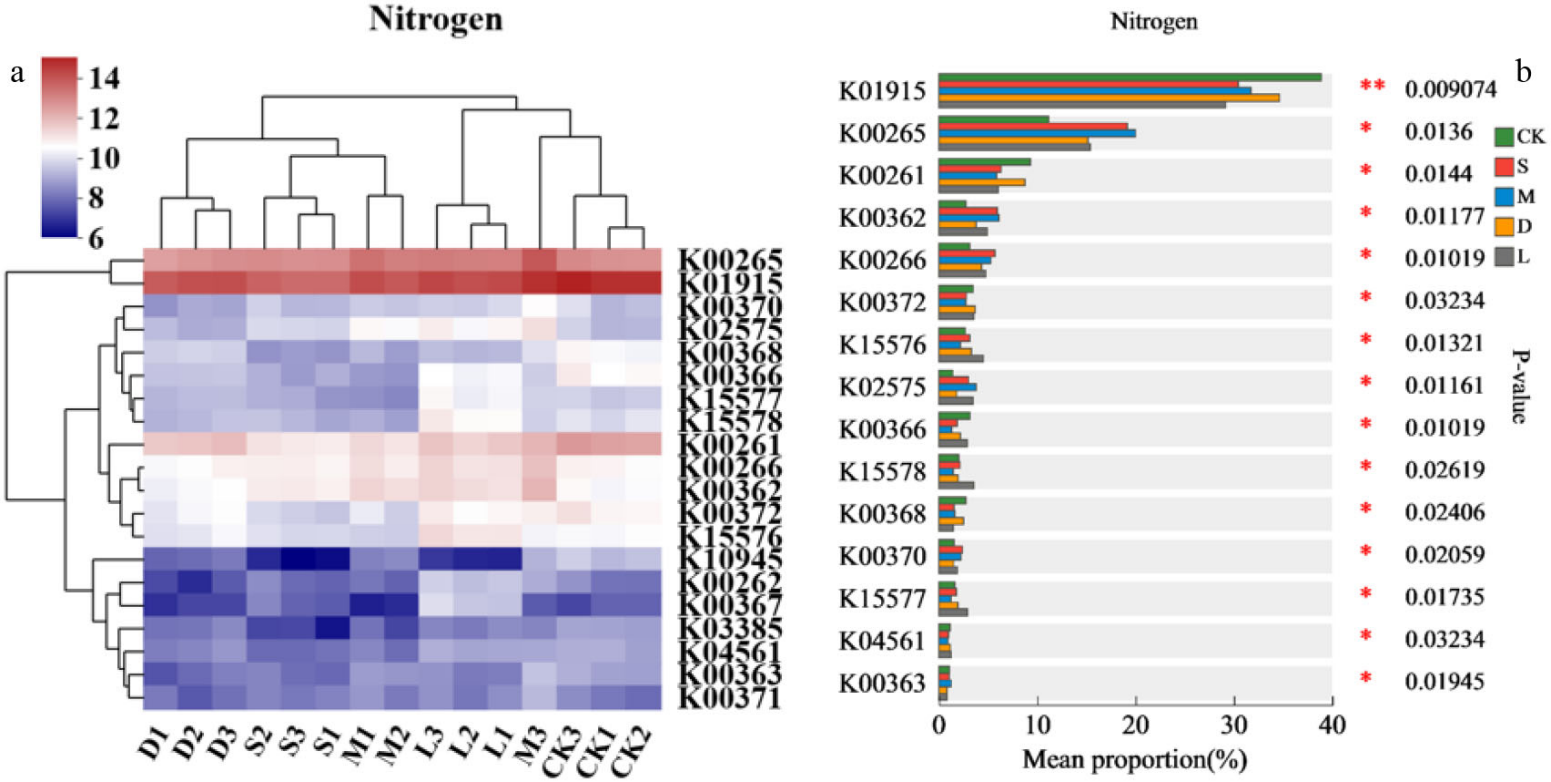
Changes in nitrogen-fixing KEGG pathways in the habitat after moss colonization. **(a)** KEGG pathways associated with the 15 most abundant genes. **(b)** KEGG pathways associated with significant differences; CK: original control soil, S: *Hyophila involuta*, M: *Eurohypnum leptothallum*, D: *Didymodon constrictus*, L: bare soil.

The nitrogen-fixing metabolic pathways in the original control soil and exposed rock surface were more similar (**Figure 7a**). Differences were noted among different species of moss. The nitrogen fixation metabolic pathways of *Hyophila involuta* are more similar to those of *Eurohypnum leptothallum*. The metabolic pathways of K00265, K00362, K00266, K02575, and K00370 in the habitat after the planting of lithophytic bryophytes on the rock surface were significantly greater than those in the original control soil, whereas those of K0195 and K00261 were significantly lower than those in the original control soil (**Figure 7b**). K15577, K15578, and K15576 pathways are significantly higher in exposed rock habitats compared with rocky moss-colonized rock surfaces and the original control soil.

#### The metabolic pathways of phosphorus metabolism microorganisms

**Figure 8:**
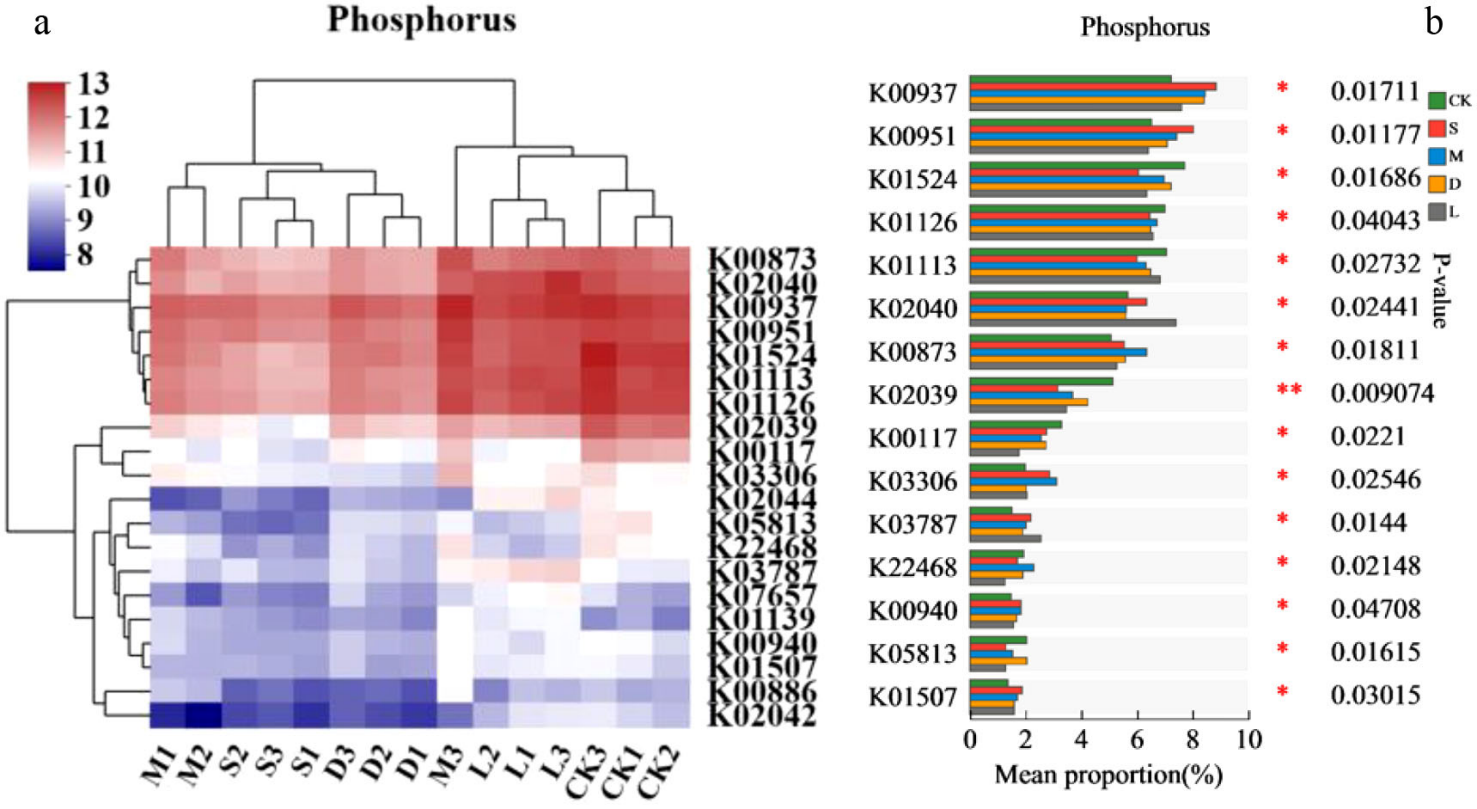
Changes in phosphorus metabolism in the habitat of lithophytic bryophytes after they were planted on rock surfaces as assessed using KEGG pathway analysis. **(a)** KEGG pathways associated with the 15 most abundant genes; **(b)** KEGG pathways associated with significant differences; CK: original control soil, S: *Hyophila involuta*, M: *Eurohypnum leptothallum*, D: *Didymodon constrictus*, L: bare soil.

The phosphorus metabolism pathways in the rock surface covered with lithophytic bryophytes were different from those in the original control soil and bare soil (**Figure 8a**). Moreover, the soil phosphorus metabolism functions of *Hyophila involuta* and *Didymodon constrictus* were similar to the original control soil and bare soil. K00937, K00951, K00873, K03306, and K01507 were significantly greater in the rock surface covered with lithophytic bryophytes than the original soil control, whereas K01524, K01113, K02039, and K00117 were significantly lower than the original soil control (**Figure 8b**).

### Different species involved in carbon fixation, nitrogen fixation, and phosphorus metabolism on rock surfaces colonized by lithophytic bryophytes

**Figure 9:**
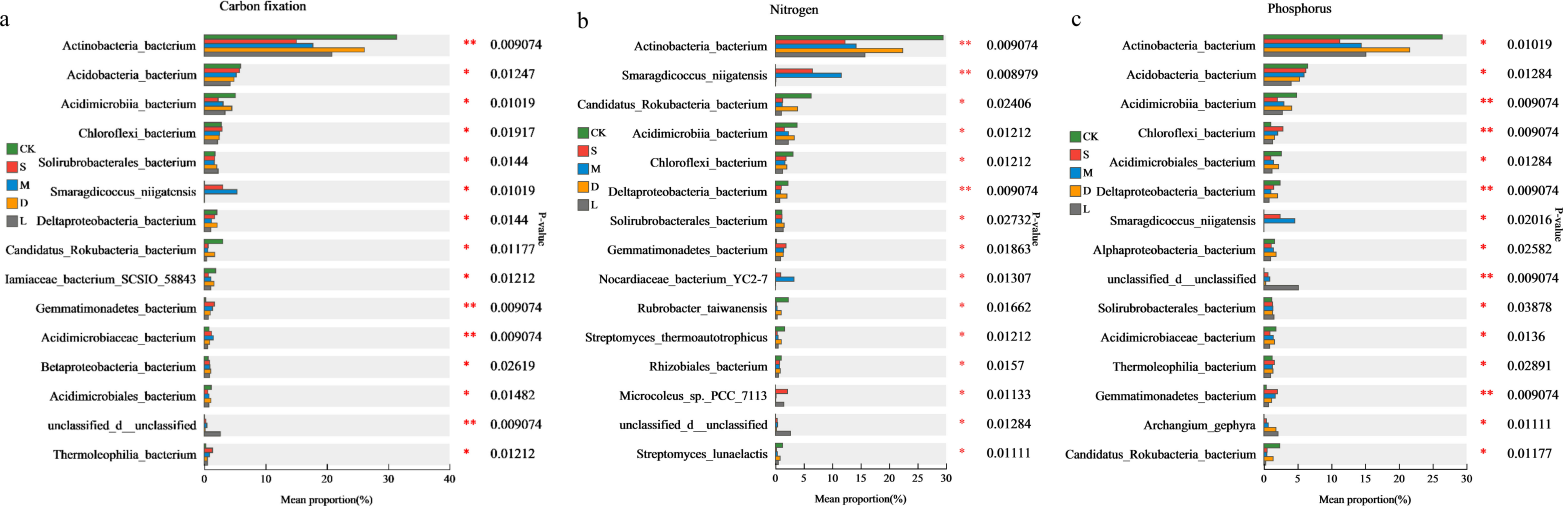
The species with significant differences between the environment of rock surface covered with bryophytes and without. **(a)** significantly different species of carbon-fixing microorganisms; **(b)** significantly different species of nitrogen-fixing microorganisms; **(c)** significantly different species of phosphorus metabolism.

There are differences in the number of microbial species with carbon fixation, nitrogen fixation, and phosphorus metabolism functions in rock surfaces covered with bryophytes compared with those in original control soil and bare soil. Among the top 15 differentiated species with carbon sequestration functions, *Smaragdicoccus_niigatensis*, *Gemmatimonadetes_bacterium*, *Acidimicrobiaceae_bacterium*, and *Thermoleophilia_bacterium* were significantly more abundant in the rock surface covered with bryophytes compared with the original control soil (**Figure 9a**). Among the top 15 differentiated species with nitrogen fixation functions, *Smaragdicoccus_niigatensis*, *Gemmatimonadetes_bacterium*, *Nocardiaceae_bacterium_YC2-7*, and *Microcoleus_sp._PCC_7113* were significantly increased in the rock surface covered with bryophytes compared with the original control soil. Among them, the abundances of *Smaragdicoccus_niigatensis* and *Nocardiaceae_bacterium_YC2–7* were increased on rock surfaces covered with *Hyophila involuta* and *Eurohypnum leptothallum* compared with *Didymodon constrictus* and bare soil (**Figure 9b**). Among the top 15 differentially expressed species related to phosphorus metabolism, *Chloroflexi_bacterium*, *Smaragdicoccus_niigatensis*, *Gemmatimonadetes_bacterium*, and *Archangium_gephyra* increased significantly in the rock surface covered with bryophytes. The abundance of *Smaragdicoccus_niigatensis* was greater in the rock surface covered with *Eurohypnum leptothallum*, and the abundance of *Hyophila involuta* than that in the rock surface covered with *Didymodon constrictus* (**Figure 9c**).

### Influence of microorganisms on soil functional traits in rocky habitats

#### The correlation between species of phosphorus metabolism and soil nutrients and organic acids

**Figure 10:**
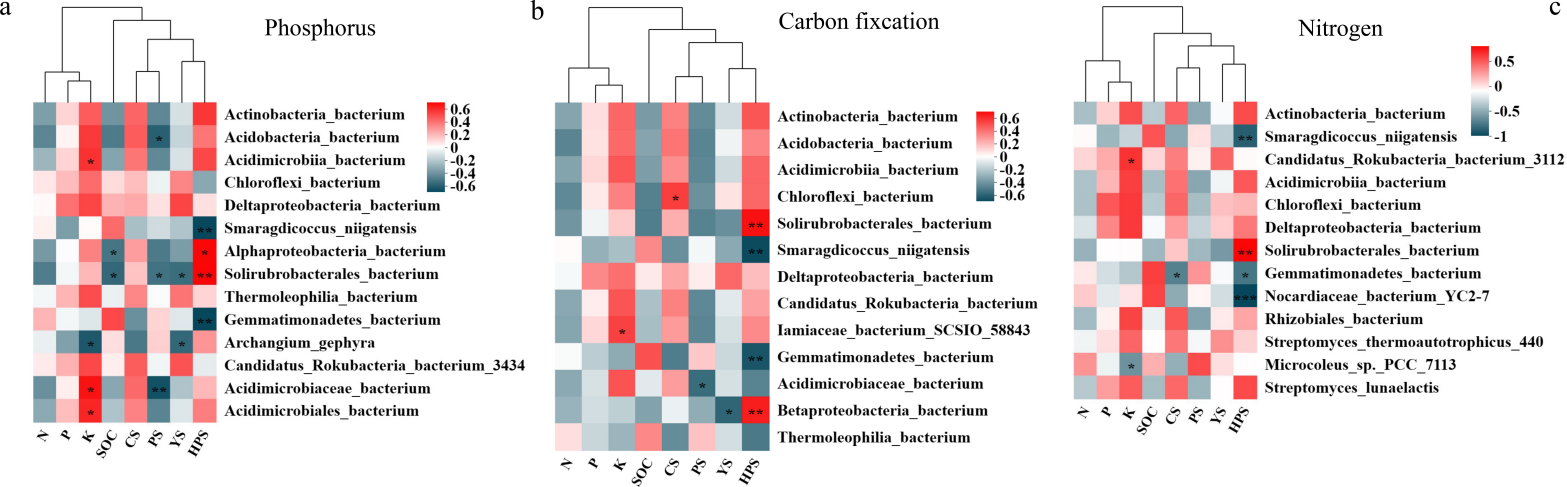
Relationships between significantly different microorganisms and soil physicochemical properties. **(a)** correlation between species related to phosphorus metabolism and soil nutrients and organic acids in the rock surface of lithophytic moss; **(b)** correlation between species related to carbon fixation and soil nutrients and organic acids, on the rock surface of lithophytic moss; **(c)** correlation between species related to nitrogen fixation and soil nutrients and organic acids, on the rock surface of lithophytic moss; N: total nitrogen; P: total phosphorus; K: total potassium; SOC soil organic carbon; CS: oxalic acid; PS: malic acid; YS: acetic acid; HPS: succinic acid.

The correlations between the microorganisms related to carbon fixation, nitrogen fixation, and phosphorus metabolism and the contents of nitrogen, phosphorus, potassium, organic carbon, oxalic acid, acetic acid, malic acid and succinic acid in rock surface soil were analyzed. We found that *Acidimicrobiia_bacterium*, *Acidimicrobiaceae_bacterium*, and *Acidimicrobiales_bacterium*, which are related to phosphorus, were significantly positively correlated with the potassium content in the soil. Moreover, *Deltaproteobacteria_bacterium* was positively correlated with the phosphorus content in the soil (**Figure 10a**). *Alphaproteobacteria_bacterium* and *Solirubrobacterales_bacterium* were significantly positively correlated with the succinic acid content in the soil and significantly negatively correlated with the soil organic carbon content. *Acidobacteria_bacterium*, *Solirubrobacterales_bacterium*, and *Acidimicrobiaceae_bacterium* were significantly negatively correlated with malic acid in the soil. *Solirubrobacterales_bacterium* and *Archangium_gephyra* were significantly negatively correlated with acetic acid in the soil.

#### The correlation between species of carbon fixation and soil nutrients and organic acids

Among the top 15 microorganisms related to carbon fixation, *Iamiaceae_bacterium_SCSIO_58843* was significantly positively correlated with the potassium content in the soil. *Gemmatimonadetes_bacterium* was positively correlated with soil organic carbon. *Chloroflexi_bacterium* was significantly positively correlated with the oxalic acid content in the soil. *Acidimicrobiaceae_bacterium* was significantly negatively correlated with malic acid in the soil. *Betaproteobacteria_bacterium* and *Solirubrobacterales_bacterium* were significantly positively correlated with succinic acid in the soil (**Figure 10b**).

#### The correlation between species of nitrogen fixation and soil nutrients and organic acids

Among the top 15 microorganisms related to nitrogen fixation, *Candidatus_ Rokubacteria_bacterium_3112* was positively correlated with the potassium content in the soil. *Microcoleus_sp._PCC_7113* was significantly negatively correlated with the potassium content in the soil. *Gemmatimonadetes_bacterium* was significantly negatively correlated with oxalic acid content. *Smaragdicoccus_niigatensis*, *Gemmatimonadetes_bacterium*, and *Nocardiaceae_bacterium_YC2–7* were significantly negatively correlated with succinic acid content in the soil. *Solirubrobacterales_bacterium* was significantly positively correlated with succinic acid (**Figure 10c**).

## Discussion

### Bryophytes increase the organic carbon and total nitrogen content in the rock surface

Bryophytes can act as ecosystem engineers regulating soil properties, and key ecosystem processes such as infiltration, nutrient cycling, and carbon (C) sequestration (Bowker et al., 2011; Delgado-Baquerizo et al., 2016; Bao et al., 2019). In this study, we found that the lithophytic bryophytes can increase the contents of soil organic carbon (SOC) and total nitrogen (TN) in rock surface habitats. A study about moss crusts in subtropical karst ecosystems showed that moss crusts can significantly increase soil organic carbon and total nitrogen content, enhance soil aggregate stability, and increase enzyme activity related to carbon and nitrogen cycling in in karst ecosystems with different degrees of degradation (Li et al., 2025). Moreover, several studies also provide evidence of bryophytes having a maximal water content higher than other poikilohydric organisms such as lichens, which implies longer hydration periods after receiving water pulses and the possibility of gaining more C through photosynthesis (Mónica Ladrón and Fernando, 2022). Bryophytes also need nitrogen (N) for their growth. However, despite a lack of developed roots and a vascular system, bryophytes can uptake N from soil and transport it to their shoots (Ayres et al., 2006; Mónica Ladrón and Fernando, 2022) and establish symbiotic associations with cyanobacteria, a group of N-fixer organisms (Mónica Ladrón and Fernando, 2022). Cyanobacteria, as symbionts in bryophytes, significantly impact the N cycle as their N-fixing activity ranges from 40% to 85% of the total fixed biologically in terrestrial ecosystems at a global scale (Rodriguez-Caballero et al., 2018; Mónica Ladrón and Fernando, 2022). Bryophytes can also enrich the soil through direct N leakage during the cyanobacterial N fixation, the decomposition of bryophyte tissues, or the phenomenon of the dehydration–rehydration process (Slate et al., 2019). Bryophytes also have a direct role by C fixation through photosynthesis. Moreover, bryophytes can act as modulators of the C cycle through their impacts, as explained, on the different forms of N and their links with other organisms. Based on the above literature analysis, bryophyte can increase the carbon and nitrogen content within the rock surface environment.

### Bryophytes reduces the total phosphorus content in rock surfaces

However, the contents of total phosphorus (TP) and total potassium (TK) were decreased in rock surfaces with lithophytic bryophytes. Inorganic P is the main P source for plants and microorganisms, and its availability is linked to the desorption and dissolution of P from soil minerals and, to a lesser extent, the decomposition of organic matter (Mónica Ladrón and Fernando, 2022). Some study indicated that the soil under the moss crust patches was predominantly composed of stable HCl-Pi, constituting 79% of the total phosphorus (Yang et al., 2025). Moreover, the moss crust patch layer exhibited lower Resin-P, NaHCO_3_-Pi, and total labile phosphorus (labile P), but higher med-labile P and stable phosphorus (stable P), compared with the lower 0–5-cm soil (Yang et al., 2025). Therefore, the bryophytes can reduce the total phosphorus but increase the organic phosphorus accumulation of soil. Moreover, coupled physicochemical and biological mechanisms drove moss effects on P cycling, ultimately through effects on soil oxygenation or reduction: Higher redox potential underlying mosses corresponded to greater microbial activity, phosphatase enzyme activity, and colonization by arbuscular mycorrhizal fungi (AMF), all of which can promote greater P availability to plants (Crowley and Bedford, 2011). Therefore, another reason for the decline about phosphorus content in rock surfaces covered with moss may be that moss absorbs phosphorus from the soil. This will provide a certain basis for improving the soil phosphorus nutrition status of karst ecosystems and the restoration of rocky desertification ecosystems. There are not true roots of bryophytes, and they rely on surface and cell wall cation exchange sites to absorb water-soluble potassium (Zhang et al., 2022). Potassium was mainly stored in the epidermis of stem and leaf and was not transported underground (Xiao et al., 2022). There are also studies indicating that bryophytes can activate insoluble potassium in soil. It was also possible that bryophytes convert insoluble potassium in soil into soluble potassium, which was absorbed by themselves, thereby reducing the decline in total potassium content in rock surfaces. However, further research will be needed on the relationship between bryophytes and soil potassium elements.

### Bryophytes prevents the loss of nutrients in rock surfaces due to soil erosion

In addition, the decline in TP and TK content was most severe in bare soil without lithophytic bryophytes covered. There is a strong consensus that bryophytes prevent both wind and water erosion (Yang et al., 2014; Bu et al., 2015) and are more effective than other biocrust constituents in doing so (Mallen-Cooper and Eldridge, 2016; Gao et al., 2020a). A study of the effects of moss patches on sediment flow loss, fluid mechanics characteristics, and surface microtopography in karst mountainous areas found that moss patches significantly reduced slope erosion rates and altered the distribution pattern of water flow (Zeng et al., 2025). In addition to physical mechanisms, changes in soil properties induced by mosses, such as increases in soil organic matter, cohesion, and fine soil texture, also protect the soil against erosive forces (Gao et al., 2020b). The rock surface without bryophytes covered can easily cause severe soil erosion, and phosphorus and potassium in the soil will also be lost along with it.

### Microorganisms in the lower of bryophyte promotes the improvement of rock surface fertility

The study found that *Acidimicrobia_Bacterium*, *Acidimicrobiaceae_Bacterium*, *Acidimicrobiales_Bacterium*, and *Iamiaceae_Bacterium_SCSIO_58843* were significantly positively correlated with the potassium content in the soil. These microorganisms are a type of acidophilic actinomycetes that can secrete citric acid, tartaric acid, oxalic acid, and acetic acid, dissolve insoluble potassium minerals in soil, generate exchangeable and water-soluble potassium, and increase the available potassium content in soil (Kurahashi et al., 2009; He et al., 2020). They can oxidize iron in trivalent iron phosphate compounds to divalent iron, release adsorbed phosphate, and increase effective phosphorus by two to five times (Cheng et al., 2022). Moreover, they can fix CO_2_ through the reverse pathway of TCA to provide organic carbon input to the soil (Poomthongdee et al., 2015). Moreover, they can convert NH_4_^+^ to NO2^−^, providing low material for denitrification and promoting nitrogen cycling (Huang et al., 2016). *Deltaproteobacteria* exhibited a significant positive correlation with phosphorus content in soil. Other bacteria were positively correlated with soil organic carbon. The bacteria can secrete phosphatases (alkaline phosphatase and acid phosphatase), catalyze the hydrolysis of organic phosphorus compounds in soil, convert organic phosphorus into inorganic phosphorus, and provide it for plants and microorganisms to absorb and utilize (Liu et al., 2023; Jia et al., 2025). After microbial death, its cellular components (nucleic acids and phospholipids) are released into the soil, becoming an important source of organic phosphorus pools and participating in the secondary cycle of phosphorus (Shu et al., 2025).

Some studies have highlighted the relevant role of the microbial communities within or below biocrusts as modulators of nutrient cycling (Liu et al., 2017; Concostrina-Zubiri et al., 2021; Qi et al., 2021). Specifically, biocrust-forming mosses can increase the abundance and diversity of bacteria and fungi beneath them (Liu et al., 2018; Maier et al., 2018). The fungi:bacteria ratio and the functional genes involved in C and N cycles also increase under moss-dominated biocrusts compared with other less-developed biocrust types (Liu et al., 2018). Mosses also interact with other members of the microbial communities in soils. The positive effects of mosses on soil stability and organic matter promote favorable microhabitats for microbial communities (Bao et al., 2019) and, therefore, boost the diversity of bacteria and fungi. This biodiversity is higher when compared with bare and cyanobacterial crusted soils (Maier et al., 2018; Tian et al., 2021). These findings suggest that an increase in these microorganisms promotes increases in potassium, phosphorus, and organic carbon contents in rock surface soil.

### Microorganisms in the lower of bryophyte promotes the improvement organic acid of rock surfaces

In this study, we found that *Alphaproteobacteria_bacterium* and *Solirubrobacterales_bacterium* were significantly positively correlated with the succinic acid content in the soil. Some studies revealed that *Solirubrobacterales_bacterium* and *Alphaproteobacteria_bacterium* can produce organic acids such as succinic acid and malic acid in TCA metabolism, which are secreted in small amounts to the extracellular environment and affect environmental pH (Jones and Santini, 2023). Moreover, some studies published that many microorganisms, such as *Enterobacter* sp. *strain 15S*, produce organic acids such as citric, fumaric, ketoglutaric, malic, and oxalic acids (Zuluaga et al., 2023). However, *Trichoderma* sp. produces different types of organic acids, including lactic acid, fuzzy acid, ascorbic acid, isocitric acid, malic acid, citric acid, and phytic acid (Bononi et al., 2020). Microorganisms produce organic acids using two methods: physiological secretion and decomposition of organic matter (Schneider et al., 2019). The organic acids produced by microorganisms chelate with cations through hydroxyl and carboxyl groups, transforming phosphates into soluble forms and increasing the effective P content (Bhattacharyya et al., 2016; Zveushe et al., 2023). Many bacteria secrete organic acids (carboxylic acids) that can increase the solubility of calcium phosphate (Jayakumar et al., 2019). In addition, organic acids can promote the dissolution of insoluble inorganic phosphate compounds, such as tricalcium phosphate, dicalcium phosphate, hydroxyapatite, and phosphate rock, thereby improving the utilization rate of phosphate fertilizers (Oteino et al., 2015; Cheng et al., 2017). Therefore, the contents of succinic acid and oxalic acid in the rock surface were affected by the presence of lithophytic bryophyte. However, further in-depth research will be needed to study the relationships between these microorganisms and organic acids.

## Conclusion

Lithophytic bryophytes can increase the number of microorganisms in rock surfaces, alter the composition and structure of the microbial communities, and enrich the species and quantities of microorganisms about carbon fixation, nitrogen fixation, and phosphorus in soil. These microorganisms can regulate the content and composition of N, P, K, and organic acids in the soil, by their physiological metabolic processes. There is a dynamic balance between bryophyte, microorganisms, and soil nutrients, which promote and constrain each other. However, this study confirmed that moss can improve soil nutrition and microbial resources on rock surfaces, and there is a correlation between these microorganisms and soil nutrients, by artificial simulation experiments. However, it is not clear which pathways these specific microorganisms affect the surface environment of rocks. This is also the research we need to carry out next.

## Data Availability

The original contributions presented in the study are included in the article/Supplementary Material. Further inquiries can be directed to the corresponding author.
